# Prevalence, treatment, and outcomes of sepsis during rapid response team calls: A systematic review and meta-analysis

**DOI:** 10.1016/j.ccrj.2026.100172

**Published:** 2026-02-25

**Authors:** Lionel Soh, Ryan Ruiyang Ling, Wei Ling Chua, Joshua Junjie Aw, Natalie Robertson, Kundan Reddy Saripalli, Deb Sharp, Sophia Zhao, Daryl Jones, Ashwin Subramaniam

**Affiliations:** a Department of Intensive Care Unit, Melbourne Health, Victoria, Australia; b Yong Loo Lin School of Medicine, National University of Singapore, National University Health System, Singapore; c Department of Anesthesia, National University Hospital, National University Health System, Singapore; d Australia New Zealand Intensive Care Research Centre, Department of Epidemiology and Preventive Medicine, Monash University, Victoria, Australia; e Alice Lee Centre for Nursing Studies, Yong Loo Lin School of Medicine, National University of Singapore, Singapore; f Singapore General Hospital, Family Medicine and Continuing Care, Duke-NUS Medical School Family Medicine Faculty, Singapore; g Department of Intensive Care Unit, Austin Health, Victoria, Australia; h Department of Respiratory and Critical Care Medicine, Changi General Hospital, Singapore; i Department of Intensive Care, Epworth HealthCare, Richmond, Victoria, Australia; j Department of Medicine, Nursing and Health Sciences, School of Nursing and Midwifery, Monash University, Melbourne, Victoria, Australia; k Department of Intensive Care, Frankston Hospital, Peninsula Health, Frankston, Victoria, Australia; l Department of Intensive Care, Victorian Heart Hospital, Monash Health, Clayton, Victoria, Australia; m Alfred Health, Melbourne, Victoria, Australia; n Austin Health, Victoria, Australia; o Department of Intensive Care Unit, Warringal Hospital, Victoria, Australia; p University of Melbourne, Department of Surgery, Department of Critical Care, Monash University, Department of Epidemiology and Preventative Medicine, Victoria, Australia; q International Society of Rapid Response Systems, Australia; r Department of Intensive Care, Dandenong Hospital, Monash Health, Dandenong, Victoria, Australia; s Peninsula Clinical School, Monash University, Frankston, Victoria, Australia; t Department of Anesthesia, Khoo Teck Puat Hospital, Singapore

**Keywords:** Sepsis, Infection, Met, Rapid response, Prevalnce

## Abstract

**Introduction:**

Sepsis is a leading cause of morbidity and mortality in hospitalised patients. Rapid Response Teams (RRTs) review clinically deteriorating patients, including those with sepsis. However, the epidemiology of sepsis in RRT calls remains unclear. This systematic review synthesised evidence on the prevalence, treatment, and outcomes of sepsis during RRT calls.

**Methods:**

Seven electronic databases (PubMed, Web of Science, Embase, CINAHL, Cochrane Library, Ovid MEDLINE, and Scopus) were searched for studies published from 1 January 2015 to 31 May 2024. All articles were independently screened and assessed for study quality using the Newcastle Ottawa Scale by two reviewers per article. The primary outcome was the prevalence of sepsis during RRT calls. Secondary outcomes included hospital mortality and length of hospitalisation. Data were pooled using random-effects meta-analyses.

**Results:**

From 5632 studies screened, 26 studies encompassing 110,909 patients and 139,076 RRT events were included. The pooled mean age was 64.4 years (95%CI: 59.2–69.7) and 48.4 % (n = 51,720, 24 studies) were male. The pooled prevalence of sepsis among all RRT calls was 23.7 % (95%CI: 15.5 %–34.6 %), with no significant difference between studies including exclusively sepsis RRT calls and studies with all causes of RRT calls (32.7 % vs. 21.8 %; p = 0.16). Common sepsis-related RRT triggers included abnormal respiratory and heart rates. Overall hospital mortality was 12.9 % (95%CI: 7.3–21.7 %) and hospital length of stay was 18 days (95%CI: 13.9–22.1), both showing no significant differences between studies including exclusively sepsis RRT calls and studies with all causes of RRT calls. New or changes in antibiotics were initiated in 38.8 % of sepsis-related RRTs. Most patients remained on the ward, while 23.3 % were transferred to the ICU.

**Conclusions:**

Sepsis is a trigger for a quarter of RRT calls, associated with substantial resource use and mortality in one eighth of patients. These findings support the need for standardised recognition protocols, escalation guidelines and prospective trials to optimise outcomes.

## 1 Background

Sepsis is a major health concern worldwide and a leading cause of morbidity and mortality in America.^1^ Sepsis is defined as a syndrome of dysregulated host response to infection, leading to life-threatening organ dysfunction.^2^ Unlike other life-threatening conditions, early sepsis syndrome can be subtle and associated with non-specific signs that are overlooked or attributed to less serious illnesses, leading to delayed recognition and treatment. The existing modern criteria for risk stratification of patients with a suspected infection include the systemic inflammatory response syndrome (SIRS)^3^ and the SOFA^4^ or the quick sequential organ failure assessment (qSOFA).^5^ As with all life-threatening conditions, early identification of sepsis is key. Studies have shown that factors reducing mortality include early recognition of sepsis and timely administration of antibiotics.^6^ Rapid response teams (RRT) provide a potential means to achieve these goals for hospitalised patients outside the Intensive Care Unit (ICU).

RRTs have been introduced into hospitals worldwide to identify, review, and treat acutely deteriorating ward patients to reduce cardiac arrests, serious adverse events, and unplanned admissions to the ICU.^7^ This typically consists of an ICU physician and a nurse who can provide critical care therapy whilst escalating at-risk patients to the relevant senior clinicians. Alongside early resuscitation with fluids, antibiotics, and vasopressors, a dedicated sepsis RRT enables a trained multidisciplinary team to recognise and implement sepsis protocols.

Despite the significant burden on the healthcare system, the epidemiology of sepsis in RRT calls remains unclear. While previous studies have reported the prevalence of sepsis (^8^,^9^)**,** these often lack detailed clinical features or biomarker profiles that could confirm sepsis as the primary reason for RRT activation. A clearer understanding of how sepsis presents during RRT calls, beyond prevalence alone, is important to improve early recognition, guide targeted management, and potentially prevent avoidable deterioration. This systematic review aimed to evaluate the literature related to the epidemiology of infections and sepsis during RRT calls. Specifically, it examined the frequency of sepsis during RRT calls and how sepsis was defined, including the use of risk stratification tools (SIRS, qSOFA, SOFA, or other) or biochemical markers. Secondary objectives included the following: presumed source of sepsis, community vs nosocomial infections, treatment approaches initiated by the RRT, patient disposition following RRT calls, and outcomes following sepsis-related RRT calls.

## 2 Methods

This systematic review was reported in accordance with the Preferred Reporting Items for Systematic Reviews and Meta-Analyses (PRISMA) Statement (Supplementary Table 2). The review was prospectively registered on PROSPERO (PROSPERO, CRD42024539594).

### 2.1 Search strategy and information sources

Seven electronic databases (PubMed, Web of Science, Embase, CINAHL, Cochrane Library, Ovid MEDLINE and Scopus) were searched from 1st January 2015 to 24th May 2024. The search strategy consisted of three concepts: “hospital rapid response team”, “sepsis”, and “infection”. Each concept's keywords, synonyms, and medical subject headings (MeSH) terms were combined using Boolean operators “OR” and “AND”. Additional citations were searched through forward and backward citation tracking (Supplementary Table 3).

### 2.2 Study selection

All articles were independently screened by two reviewers, first by title and abstract, followed by full-text review, with a total of eight reviewers involved. Screening was conducted using COVIDENCE^10^ and any discrepancy was adjudicated by a third reviewer. Articles were included if they: (i) were cohort studies (prospective and retrospective), randomized control trials, or case–control studies published between 1st Jan 2015 and 24th May 2024; (ii) involved adult Patients (≥18 years) in general wards who had a RRT activation (nomenclature of RRT: medical emergency team, critical care outreach team, patient at-risk team); and (iii) reported on at least 20 adult patients.

### 2.3 Eligibility criteria

We assessed all the relevant studies, and their citation lists to identify articles for inclusion that may have been missed during the literature search. The studies were excluded if they were based solely on sepsis response teams. Studies were also excluded if they were not original research (e.g. review articles, expert opinions, letters, and commentaries); if they were unpublished, conference abstracts, or non-peer-reviewed, not in the English language, or if full-text articles were not available. We also excluded any case reports and series (≤20 participants) to avoid publication bias.

### 2.4 Data extraction

Data were extracted by four reviewers (LS, SZ, JA, KRS), with each article independently reviewed by two reviewers using a prespecified data extraction form. Discrepancies were resolved through consensus or discussions with a senior reviewer (AS). Extracted data included study characteristics (author first name, year of publication, study design, study period, sample size, and country where the study was conducted, and type of study), patient demographic data, clinical characteristics, and clinical outcomes using a pre-specified datasheet. The final tabulated data were verified by RRL and the senior author (AS).

### 2.5 Quality assessment and risk of bias in individual studies

Each included articles was independently appraised by two reviewers using the Newcastle Ottawa Scale (NOS^11^) to assess the risk of bias for observational studies (Supplementary Table 6). Studies scoring fewer than 4 out of 9 points were classified as having a high risk of bias. Studies scoring 4–6 were rated as ‘fair’, and studies scoring 7 or above were rated as ‘good’. Any discrepancies from the NOS were reviewed and resolved by the senior author (AS). In addition, Grading of Recommendations, Assessment, Development, and Evaluations (GRADE) was used to determine the certainty of evidence.

### 2.6 Study outcomes

The primary outcome was the prevalence of patients who had a diagnosis of sepsis (which was defined using qSOFA, SOFA, SIRS or via clinical judgement) during an RRT call. The secondary outcomes included the associated hospital mortality and hospital length of stay. The tertiary outcomes included the presumed source of sepsis, triggers for RRTs, location of infection (hospital vs. community), the prevalence of patients with community-acquired vs nosocomial sepsis, the interventions at the time of RRT review, and patients' disposition at the end of RRT review. We evaluated studies that included exclusively sepsis-related RRT calls, as well as studies that included RRT calls from all causes.

### 2.7 Data analysis

**Fig. 1:**
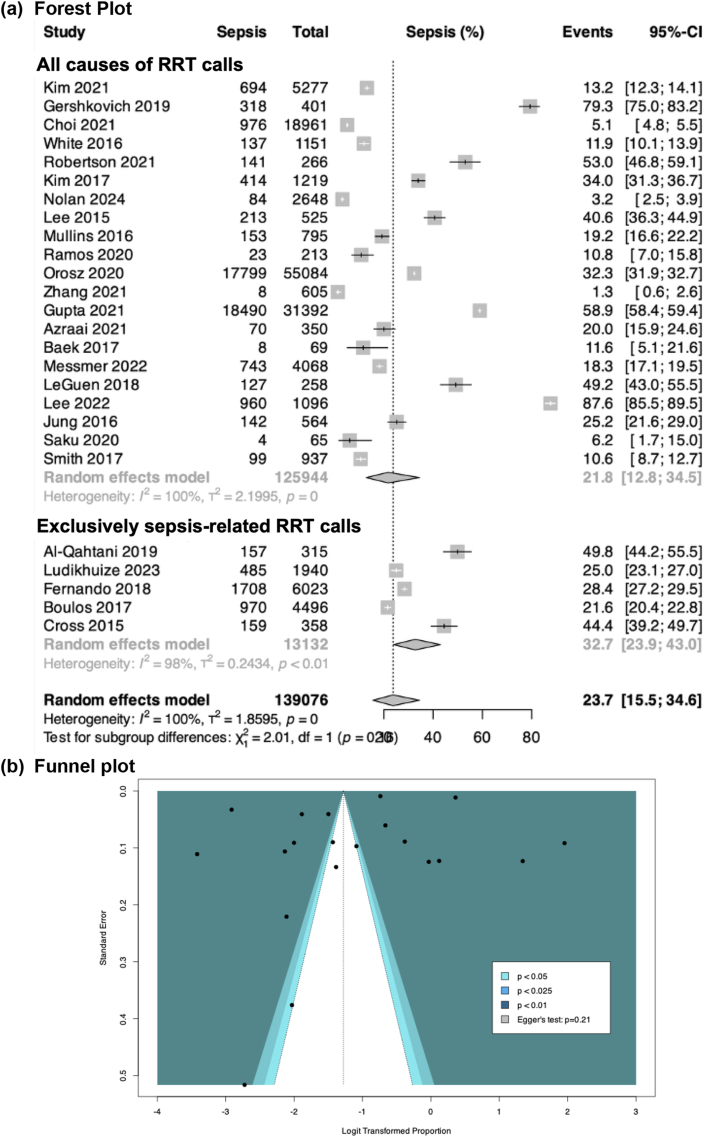
The pooled proportion of sepsis-related RRT calls, presented as overall and stratified by study type.

Meta-analyses were conducted using R version 4.0.1. For studies with overlapping participant data, only the larger study was included in the primary meta-analysis. Random-effects meta-analyses (DerSimonian and Laird method[12], [13], [14]) were performed using the logit transformation, with 95 % CIs calculated via the Clopper-Pearson method.^15^ Binary outcomes were reported as pooled proportions or odds ratios if comparative data were available, while continuous outcomes were reported as pooled means or mean differences (Fig. 1). Where there were zero values in a comparative meta-analysis of binary outcomes, a continuity correction factor of 0.5 was applied to facilitate analyses. When only medians and interquartile ranges were provided, means and standard deviations were estimated using established formulas as per Wan et al..^16^^,^^17^

Sensitivity analyses were conducted, excluding studies with poor quality (NOS score <4), to assess the impact of intra-study risk of bias on the reporting of sepsis during an RRT call. Publication bias was assessed by visual inspection of funnel plots and Egger's test. As *I*^*2*^ statistics tend to overestimate inter-study heterogeneity, we assessed the heterogeneity as part of the GRADE^18^ approach, accounting for both quantitative heterogeneity (using *I*^2^ statistics, exploring for sources of heterogeneity using subgroup analysis) and qualitative heterogeneity (distribution of the point estimates and degree of overlap of the 95%CIs) of studies in the forest plots. In addition, we analysed to stratify studies using SOFA-based (Sepsis-3-aligned) definitions of sepsis versus those with more traditional definitions. Finally, we conducted an analysis comparing studies including exclusively septic RRT calls to studies with all causes of RRT calls. A two-sided p-value <0.05 was considered to indicate significance in our analysis.

## 3 Results

### 3.1 Included studies

The search yielded 5632 studies after removing duplicates. Following title-abstract screening, 52 studies underwent full-text reviews. Finally, 26 studies[19], [20], [21], [22], [23], [24], [25], [26], [27], [28], [29], [30], [31], [32], [33], [34], [35], [36], [37], [38], [39], [40], [41], [42], [43], [44] were included (Supplementary Table 1).

### 3.2 Study characteristics

The 26 studies included 110,909 patients with 139,076 documented RRT events. Of these, 24 were observational studies (6 prospective (22, 23, 26,27, 35, 42), 18 retrospective (19–21,24, 25, 28–34,36, 38, 39,41, 43, 44) and two were case–control studies (Table 1) (37,40). Studies originated from nine countries, predominantly from Australia (n = 12) (22, 23, 25,28–30,32, 35, 39,41, 43, 44), followed by South Korea (n = 6) (19, 21, 24,26, 33, 36), Canada (n = 2) (20,42), and one each from Finland, France, New Zealand, the USA, Saudi Arabia, and Switzerland (27, 31, 34,37, 38, 40). Twenty studies were single-centre (21–28,30–36,38, 39, 41,43,44) and six were multicentre studies (19, 20, 29,37,40).

**Table 1:** Characteristics of included studies.

First Author, Year of publication	Country	Single/Multicenter, Type of study	Definition of sepsis	Total number of patients	Total RRTs	Male	Age Mean [SD] years
Studies including all causes of RRTs calls							
Kim 2021	South Korea	Multicenter, retrospective	qSOFA; clinical	5277	5277	3210	62.9 [14.4]
Gershkovich 2019	Canada	Multicenter, retrospective	qSOFA; SIRS	401	401	254	61.6 [15.3]
Choi 2021	Korea	Single, retrospective	SOFA	973	18961	594	61.5 [13.5]
White 2016	Australia	Single, retrospective	Clinical	800	1151	468	61.3 [12.8]
Robertson 2021	Australia	Single, prospective	Clinical	381	266	249	55.3 [2.2]
Kim 2017	South Korea	Single, retrospective	Clinical	1219	1219	795	60.5 [3.1]
Nolan 2024	Australia	Single, retrospective	qSOFA	2084	2648	114	64.3 [4.9]
Lee 2015	South Korea	Single, prospective	SOFA	525	525	348	58.0 [3.3]
Mullins 2016	New Zealand	Single, prospective	Clinical	630	795	–	64.0
Ramos 2020	Australia	Single, retrospective	Clinical	213	213	58	77.8 [3.8]
Orosz 2020	ANZ	Multicenter, retrospective	qSOFA; SOFA	55084	55084	29405	65.4 [16.9]
Zhang 2021	Australia	Single, retrospective	Clinical	605	605	202	78.8 [3.1]
Gupta 2021	US	Single, retrospective	Clinical	12,122	31392	–	–
Azraai 2021	Australia	Single, retrospective	Clinical	487	721	248	44.8 [19.3]
Baek 2017	Seoul, Korea	Single, retrospective	SOFA	69	69	–	33.6 [4.3]
Messmer 2022	Switzerland	Single, retrospective	Clinical	4068	4068	2067	65.9 [15.7]
LeGuen 2018	Australia	Single, prospective	qSOFA	193	258	88	73.5 [4.8]
Lee 2022	South Korea	Single, retrospective	SOFA	1096	1096	675	67.4 [14.7]
Jung 2016	France	Multicenter, case-control	Clinical	19073	564	10194	59.0 [18.0]
Saku 2020	Finland	Single, retrospective	Clinical	61	65	21	70.5 [9.9]
Smith 2017	Australia	Single, retrospective	SIRS	700	937	496	67.5 [3.8]

Studies including exclusively sepsis-related RRT calls							

Al-Qahtani 2019	Saudi Arabia	Multicenter, case-control	SOFA	245	315	172	–
Ludikhuize 2023	Australia	Single, retrospective	qSOFA	1940	1940	622	70.8 [3.8]
Fernando 2018	Canada	Multicenter, prospective	SOFA; SIRS	1708	6023	963	65.0
Boulos 2017	Australia	Single, retrospective	qSOFA; SIRS	646	4496	335	68.5 [17.4]
Cross 2015	Australia	Single, retrospective	SIRS	309	358	142	71.7 [3.8]
**Total**	**-**	**-**	**-**	**110,909**	**139,076**	**51720 (48.4 %)**	

qSOFA – quick Sequential Orga, RRT = Rapid Response Team, SD = Standard Deviation SIRS = Systemic Inflammatory Response Syndrome.

Eighteen studies were rated as good quality (19–21,23–25,28–31,33–35,37, 39, 40,43,44) and eight as fair quality (22, 26, 27,32, 36, 38,41,42). The overall mean age ranged between 33.6 and 77.8 years, with a pooled mean of 64.4 years (95%CI: 59.2–69.7). Across 24 studies, 48.4 % of patients (n = 51,720) were male. Thirty-five percent of RRT calls occurred after hours (11 studies) (20, 22, 25,29, 31, 34,38,41–44), and 26.4 % in the weekend (2 studies) (22,29).

### 3.3 Primary outcome: prevalence of sepsis in rapid response calls

**Fig. 2:**
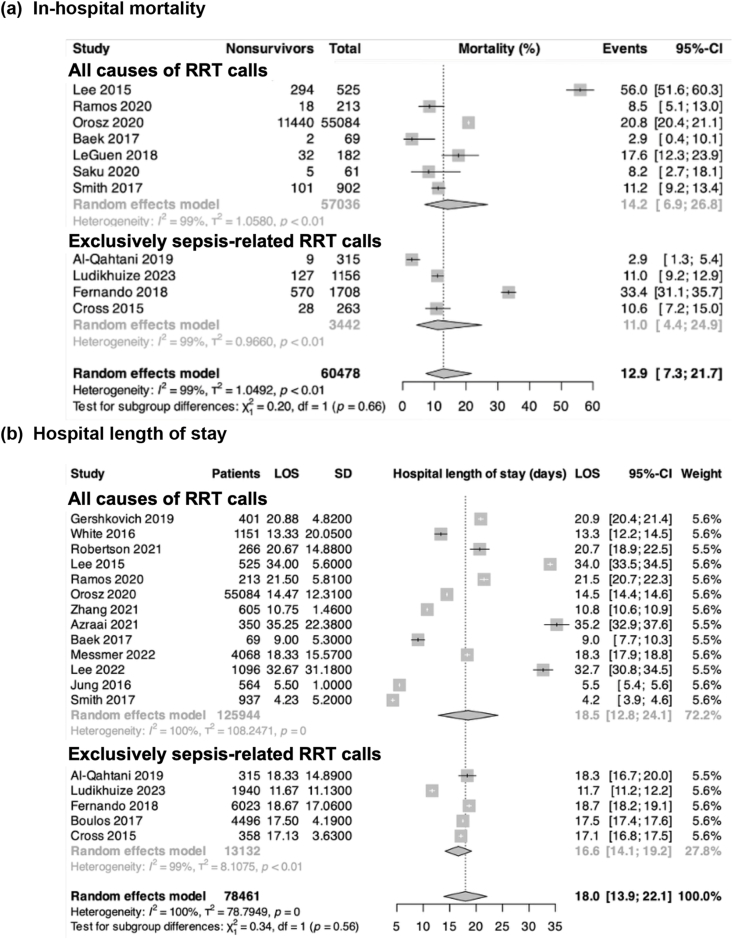
Secondary outcomes: (a) in-hospital mortality; and (b) hospital length of stay after sepsis-related RRT calls according to study type.

Of the included studies, five exclusively examined sepsis-related RRT calls,[40], [41], [42], [43], [44] while 21 evaluated RRT calls from all causes.[19], [20], [21], [22], [23], [24], [25], [26], [27], [28], [29], [30], [31], [32], [33], [34], [35], [36], [37], [38], [39] Overall, 32.4 % of the patients (n = 45,082) had a sepsis-related RRT event (Supplementary Tables 4 and 5). Across 26 studies, the pooled prevalence of sepsis among RRT calls was 23.7 % (95%CI: 15.5 %–34.6 %; moderate certainty, p_egger_ = 0.21, no publication bias) (Fig. 2).

When stratified by study type, the prevalence of sepsis was 21.8 % (95%CI: 12.8 %–34.5 %) for studies that included all causes of RRTs and 32.7 % (95%CI: 23.9 %–43.0 %) for studies that included exclusively sepsis-related RRT calls; however, the difference was not statistically significant (p = 0.16). As no studies were rated of poor quality, sensitivity analysis excluding low-quality studies was not performed (Supplementary Table 5).

**Fig. 3:**
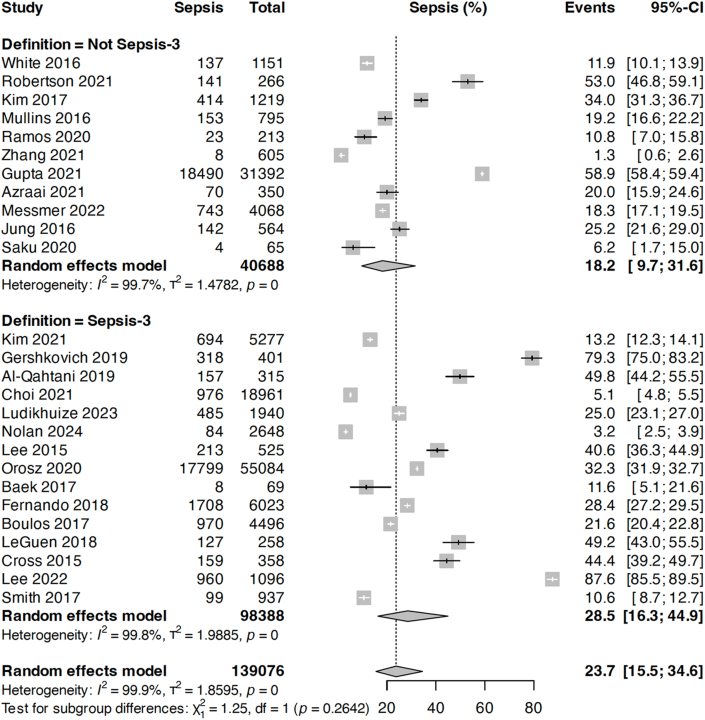
Prevalence of sepsis-related RRT calls stratified according to Sepsis-3-related definitions compared with other (Non-Sepsis-3-related) sepsis definitions.

When studies were stratified according to sepsis definition, we found that the pooled prevalence of sepsis during RRT calls was 28.5 % compared with 18.2 % for those using more traditional definitions (p = 0.26; Fig. 3).

Respiratory rate and heart rate abnormalities were the most common triggers for sepsis-related RRT calls, while hypoxia, hypotension, change in GCS and clinical concern were common triggers for RRT calls not related to sepsis (Table 2; Supplementary Table 7).

**Table 2:** Pooled comparison based on sepsis-related and non-sepsis-related rapid response team triggers.

Trigger	Non-sepsis-related RRT calls	Sepsis-related RRT calls
Airway	0.2 % (0.02 %–1.2 %)	0.1 % (0.01 %–1.7 %)
Respiratory rate	11.0 % (5.4 %–21.1 %)	18.1 % (6.8 %–40.2 %)
Hypoxia	9.6 % (4.0 %–21.3 %)	0.4 % (0.01 %–17.9 %)
Heart rate	9.3 % (2.9 %–25.7 %)	17.6 % (4.7 %–48.1 %)
Hypotension	21.3 % (5.4 %–56.0 %)	4.5 % (0.8 %–22.3 %)
Change in GCS	9.5 % (3.0 %–26.4 %)	2.5 % (0.5 %–11.0 %)

GCS – Glasgow Coma Scale.

### 3.4 Secondary outcomes

*In-hospital mortality:* Eleven studies reported on hospital mortality: seven studies including all causes of RRT calls (26, 28, 29,33, 35, 38,39) and four studies including exclusively septic RRT calls (40–42,44). The pooled hospital mortality was 12.9 % (95%CI: 7.3–21.7 %). The pooled hospital mortality was 11.0 % (95%CI: 4.4 %–24.9 %; high certainty) for studies exclusively including septic RRT calls and 14.2 % for studies with all causes of RRTs (95%CI: 6.9 %–26.8 %; high certainty), with no statistically significant difference between the two groups (Fig. 2a–Supplementary Table 5).

*Hospital length of stay:* Overall, 18 studies reported on hospital length of stay: 13 for studies including all causes of RRT calls (20, 22, 23,26,28–30,32–34,36, 37, 39) and five for studies exclusively including septic RRT calls[40], [41], [42], [43], [44]). The pooled mean hospitalisation was 18.0 days (95%CI: 13.9–22.1). The pooled hospital length of stay was 16.6 days (95%CI: 14.1–19.2 days; moderate certainty) for studies exclusively including septic RRT calls and 18.4 days (95%CI: 12.8–24.1 days; high certainty) for studies with all causes of RRTs (Fig. 2b–Supplementary Table 5).

### 3.5 Tertiary outcomes

A summary of the tertiary outcomes is presented in Table 3 and Supplementary Tables 8–11.

**Table 3:** Pooled estimates of infection source, interventions, and patient disposition during rapid response team calls, with GRADE certainty ratings.

Outcome	Studies	Point Estimate (95%CI)	GRADE Certainty
Source of infection			
- Pulmonary	9	34.6 % (25.3 %–45.3 %)	Moderate^a^
- Skin	8	0.1 % (0.003 %–3.3 %)	Moderate^b^
- Urinary tract infections	8	11.4 % (9.0 %–14.3 %)	High
- CNS	8	0.03 % (0.003 %–2.4 %)	Moderate^b^
- Abdominal	8	13.1 % (3.5 %–38.5 %)	Moderate^b^
- Others	8	3.6 % (0.4 %–25.0 %)	Low^a^^,^^b^

Location of infection			

- Hospital	2	30.4 % (8.5 %–67.2 %)	Low^a^^,^^b^
- Community	3	86.7 % (9.4 %–99.8 %)	Low^a^^,^^b^

Intervention(s) during RRT review			

- Antibiotics	4	38.8 % (21.9 %–58.8 %)	Low^a^^,^^b^
- Vasopressors	2	19.9 % (10.0 %–35.9 %)	Low^a^^,^^b^
- Intubation	2	16.2 % (10.1 %–25.0 %)	Low^a^^,^^b^

Patient disposition at the end of a RRT review			

- Stay in ward	13	69.2 % (60 %–77.1 %)	Moderate^a^
- ICU/HDU	22	23.3 % (12.4 %–39.4 %)	Moderate^b^
- Emergency surgery	5	1.7 % (0.9 %–2.9 %)	Moderate^b^
- Died during MET	3	1.7 % (0.8 %–3.7 %)	Low^a^^,^^b^

Abbreviations: ICU – intensive care unit, HDU – high dependency unit, RRT – Rapid Response team, CI – confidence interval, GRADE - Grading of Recommendations, Assessment, Development, and Evaluations.

a Downgrade for inconsistency.

b Downgrade for imprecision.

*Source of infections:* This was reported for all studies. The most common sources were respiratory (9 studies (20, 21, 23,35, 39, 40,42–44): 34.6 % 95%CI: 25.3 %–45.3 %; moderate certainty) and intra-abdominal (8 studies (20, 21, 35,39, 40, 42–44): 13.1 % 95%CI: 3.5 %–38.5 %; moderate certainty). The least common source was from the CNS (8 studies (20, 21, 35,39, 40, 42–44): 0.03 % 95%CI: 0.003 %–2.4 %; low certainty).

*Community vs hospital-acquired:* Three studies (25, 43, 44) reported whether infections were acquired from a community or a nosocomial source. The infections acquired from the community were relatively higher (86.7 %; 95%CI: 9.4 %-99.8; low certainty), when compared with hospital-acquired (30.4 %; 95%CI: 8.5 %–67.2 %; low certainty).

*Intervention during RRT review:* Four studies (40, 41, 43,44) examined the type of interventions utilised at RRT calls. The pooled rate of new/change in antibiotics occurred in 38.8 % (34.6 % 95%CI: 21.9 %–58.8 %; low certainty). Patients were administered vasopressors in 19.9 % [95 % CI: 10.0 %–35.9 %; low certainty], and the pooled rate of intubation happened in 16.2 % (95%CI: 10.1 %–25.0 %; low certainty).

*Disposition of RRT calls:* This was reported for all studies. Most patients remained in the same ward (13 studies (24,31–36,38–41): 69.2 %; 95%CI: 60.7 %–77.1 %; moderate certainty), while a quarter of the patients were admitted to ICU (22 studies (20–24,26,28–43): 23.3 %; 95%CI: 12.4 %–3.7 %; moderate certainty). Patients undergoing surgery for source control or dying during an RRT call were poorly reported.

## 4 Discussion

### 4.1 Key findings

This systematic review synthesized data from 26 studies involving over 110,000 patients and 139,000 RRT events. To our knowledge, this is the first review to evaluate published data on the prevalence of sepsis during RRT calls and to examine outcomes specific to sepsis-related RRT calls. Several key findings emerged. First, almost one in four RRTs were related to sepsis (pooled prevalence 23.7 %), and these calls occurred in a patient cohort with a mean age of 64 years. Although few studies focused exclusively on sepsis RRT calls, the pooled prevalence did not differ significantly between studies including exclusively septic RRT calls and those including all causes of RRT calls. The RRT triggers were different for sepsis-related RRT calls, notably changes in respiratory and heart rates, while hypoxia, hypotension, changes in GCS, and clinical concern were more typical in RRT calls not related to sepsis. Second, the pooled hospital mortality for patients with sepsis in RRT events was 12.9 % and the pooled hospital length of stay was 18 days, with no significant differences between sepsis-related and non-sepsis-related RRT calls for either outcome. Third, the initiation or change in antibiotics occurred in approximately 40 % of sepsis-related RRT calls. While most patients remained on the ward following a RRT review remained on the ward, nearly one-quarter required transfer to the ICU. These findings highlight the clinical burden of sepsis in RRT scenarios and support the need for standardized sepsis recognition protocols and prospective evaluation of targeted interventions to improve outcomes.

### 4.2 Comparison with published literature

Sepsis represents a substantial proportion of rapid response system activations, accounting for nearly 24 % of all RRT calls.^41^ Despite this high burden, few studies have specifically examined sepsis-triggered RRT calls. There was a wide range of sepsis prevalence in RRT calls reported in the examined studies, with significant heterogeneity in the reporting of sepsis. A possible explanation is the differing sepsis screening tools utilized by clinicians. SIRs, qSOFA and SOFA scores are commonly employed by clinicians in predicting sepsis and evaluating mortality for patients with sepsis. Given the differing criteria for these scoring systems, it is unsurprising that there is a spectrum of prevalences reported. The pooled prevalence in this systematic review was at least consistent with what is reported in the literature (8,9). This highlights the need for greater specificity in how sepsis is identified and reported in the context of rapid response systems. Moreover, while common physiological triggers such as changes in respiratory and heart rate were prominent in sepsis-related RRTs, consistent with the sequelae of evolving sepsis in patients, hypoxia and altered mental status were more common in RRT calls not related to sepsis, pointing to differences in how sepsis may initially manifest and be recognized. An area that may facilitate the early detection of sepsis is the integration of AI in electronic health systems to flag deteriorating patients in wards.

The findings related to outcomes are equally important. Overall hospital mortality among patients with sepsis-related RRTs was approximately 13 %, and hospital length of stay averaged 18 days, with no significant differences between sepsis-related and non-sepsis-related RRT calls. These findings were consistent with previous studies for both mortality and duration of hospitalisation.^45^^,^^46^ This suggests that despite targeted interventions, sepsis remains a high-risk condition with consistent adverse outcomes, regardless of the primary reason for the RRT activation. This is further evidence that sepsis has an increasing burden on the healthcare system, especially in older and multimorbid patients. Therefore, early recognition of sepsis is essential.

An interesting finding in the interventions employed at RRTs was the relatively low rate of antibiotic changes or introductions for deteriorating patients. Du et al. demonstrated that the introduction of antibiotics in patients with sepsis in RRT calls was close to 60 % and as high as 81.3 % if a pharmacist was present.^47^ There may be several reasons for the relatively low rate of antibiotic modification or commencement. First, there may have been operational differences in the RRT, including variability in RRT team composition, response protocols, or thresholds for escalation of care, raising questions about consistency in management practices. Alternatively, the relatively low frequency of changes of antibiotics may reflect that the patient was thought to be on appropriate treatment before the RRT, but that sufficient time may not have elapsed for them to be effective. Another possible explanation is the delayed recognition of sepsis by the responding RRT or ward clinicians. Clinically, these findings have several implications. There is an urgent need to develop and implement standardised sepsis recognition protocols within RRS frameworks to ensure timely and appropriate interventions.^41^ Hospitals may also benefit from stratifying RRT responses based on infection risk or using predictive algorithms to improve sepsis detection. Additionally, training programs aimed at improving early sepsis recognition and response could reduce variability and improve outcomes. Finally, prospective studies are warranted to test targeted intervention bundles, combining rapid diagnostics, early antimicrobials, and physiological monitoring, to determine their effect on patient-centred outcomes in sepsis-related RRT events.

We found that the mortality and hospital length of stay for RRT patients with sepsis were similar to those of non-septic patients. These findings are likely to be confounded by the presence of chronic co-morbidity, baseline illness severity, timing of recognition of deterioration, the presence (or absence) of limitations of medical treatment, and selection bias.

## 5 Strengths and limitations

This study had several strengths. To our knowledge, this is the largest and most comprehensive systematic review assessing the prevalence of sepsis in RRTs and its associated outcomes. There were no ‘poor’ quality studies as assessed by the NOS system that were included in this review. Finally, the GRADE framework was used to assess the certainty of evidence and the presence of any residual confounding selection bias and therefore strengthening of recommendations.

There were several limitations of this systematic review. First, there was significant heterogeneity across studies in terms of design, patient populations, definitions of sepsis, and reporting of outcomes, which limits the generalizability and precision of pooled estimates. However, we have provided a separate analysis based on sepsis-3-aligned definitions and did not find major differences based on the sepsis definition. Secondly, most studies did not report detailed criteria for RRT activation or sepsis diagnosis, making it difficult to compare interventions or triggers consistently across studies. Third, data on important confounders, such as comorbidities, severity of illness, and antimicrobial resistance, were often missing or inconsistently reported. Fourth, although publication bias was not significant on Egger's test, the presence of small-study effects and underreporting of negative findings cannot be ruled out. Fifth, most studies did not include a unique patient identifier in RRTs. This would mean that patients with repeated RRTs may have their data included multiple times. Six, many studies lacked granular data on the timing of interventions, specific treatments administered, and long-term patient-centred outcomes such as quality of life or functional recovery, highlighting the need for more comprehensive, prospective evaluations of sepsis in the rapid response context. Our study excluded cohorts of <20 patients as we believed they would be prone to bias. Inclusion of such studies may have made small quantitative differences to our findings. Finally, all the included studies were conducted in high-income countries and in English, which limits the generalizability of findings to low- and middle-income countries and those with a spoken language other than English.

## 6 Conclusion

This systematic review demonstrates that sepsis is a common trigger for RRT calls, accounting for nearly one in four events. Although mortality and hospital length of stay were similar between sepsis-related and non-sepsis-related RRT calls, the burden of sepsis remains substantial. Significant heterogeneity in definitions and reporting exists. Given the high prevalence of sepsis, our findings underscore the urgent need for standardised sepsis identification protocols and prospective studies to evaluate targeted interventions that can enhance patient outcomes within the context of rapid response systems.

## Declaration of competing interest

The authors declare the following financial interests/personal relationships that may be considered as potential competing interests: Dr Ashwin Subramaniam is an associate editor for Critical Care and Resuscitation If there are other authors, they declare that they have no known competing financial interests or personal relationships that could have appeared to influence the work reported in this article.
