# Supplementary material for: Prevalence, treatment, and outcomes of sepsis during rapid response team calls: A systematic review and meta-analysis
Source: Crit Care Resusc. 2026 Feb 25;28(1):100172. doi: 10.1016/j.ccrj.2026.100172 (PMC12955560; doi:10.1016/j.ccrj.2026.100172)
Supplement: Multimedia component 1 [file mmc1.docx]

**Supplementary Table 1:** PRISMA flowchart

**Supplementary Table 2:** PRISMA checklist

| **Section and Topic** | **Item #** | **Checklist item** | **Reported on page #** |
| --- | --- | --- | --- |
| **TITLE** | | |  |
| Title | 1 | Identify the report as a systematic review. | 1 |
| **ABSTRACT** | | |  |
| Abstract | 2 | See the PRISMA 2020 for Abstracts checklist. | 2 |
| **INTRODUCTION** | | |  |
| Rationale | 3 | Describe the rationale for the review in the context of existing knowledge. | 3 |
| Objectives | 4 | Provide an explicit statement of the objective(s) or question(s) the review addresses. | 4 |
| **METHODS** | | |  |
| Eligibility criteria | 5 | Specify the inclusion and exclusion criteria for the review and how studies were grouped for the syntheses. | 4 |
| Information sources | 6 | Specify all databases, registers, websites, organisations, reference lists and other sources searched or consulted to identify studies. Specify the date when each source was last searched or consulted. | 5 |
| Search strategy | 7 | Present the full search strategies for all databases, registers and websites, including any filters and limits used. | 5 |
| Selection process | 8 | Specify the methods used to decide whether a study met the inclusion criteria of the review, including how many reviewers screened each record and each report retrieved, whether they worked independently, and if applicable, details of automation tools used in the process. | 5 |
| Data collection process | 9 | Specify the methods used to collect data from reports, including how many reviewers collected data from each report, whether they worked independently, any processes for obtaining or confirming data from study investigators, and if applicable, details of automation tools used in the process. | 5 |
| Data items | 10a | List and define all outcomes for which data were sought. Specify whether all results that were compatible with each outcome domain in each study were sought (e.g. for all measures, time points, analyses), and if not, the methods used to decide which results to collect. | 5 |
|  | 10b | List and define all other variables for which data were sought (e.g. participant and intervention characteristics, funding sources). Describe any assumptions made about any missing or unclear information. | 5 |
| Study risk of bias assessment | 11 | Specify the methods used to assess risk of bias in the included studies, including details of the tool(s) used, how many reviewers assessed each study and whether they worked independently, and if applicable, details of automation tools used in the process. | 5 |
| Effect measures | 12 | Specify for each outcome the effect measure(s) (e.g. risk ratio, mean difference) used in the synthesis or presentation of results. | 6 |
| Synthesis methods | 13a | Describe the processes used to decide which studies were eligible for each synthesis (e.g. tabulating the study intervention characteristics and comparing against the planned groups for each synthesis (item #5)). | 6 |
|  | 13b | Describe any methods required to prepare the data for presentation or synthesis, such as handling of missing summary statistics, or data conversions. | 6 |
|  | 13c | Describe any methods used to tabulate or visually display results of individual studies and syntheses. | 6 |
|  | 13d | Describe any methods used to synthesize results and provide a rationale for the choice(s). If meta-analysis was performed, describe the model(s), method(s) to identify the presence and extent of statistical heterogeneity, and software package(s) used. | 6 |
|  | 13e | Describe any methods used to explore possible causes of heterogeneity among study results (e.g. subgroup analysis, meta-regression). | 6 |
|  | 13f | Describe any sensitivity analyses conducted to assess robustness of the synthesized results. | 6 |
| Reporting bias assessment | 14 | Describe any methods used to assess risk of bias due to missing results in a synthesis (arising from reporting biases). | 5 |
| Certainty assessment | 15 | Describe any methods used to assess certainty (or confidence) in the body of evidence for an outcome. | 5 |
| **RESULTS** | | |  |
| Study selection | 16a | Describe the results of the search and selection process, from the number of records identified in the search to the number of studies included in the review, ideally using a flow diagram. | 7 |
|  | 16b | Cite studies that might appear to meet the inclusion criteria, but which were excluded, and explain why they were excluded. | 7 |
| Study characteristics | 17 | Cite each included study and present its characteristics. | 7 |
| Risk of bias in studies | 18 | Present assessments of risk of bias for each included study. | 7 |
| Results of individual studies | 19 | For all outcomes, present, for each study: (a) summary statistics for each group (where appropriate) and (b) an effect estimate and its precision (e.g. confidence/credible interval), ideally using structured tables or plots. | 7-9 |
| Results of syntheses | 20a | For each synthesis, briefly summarise the characteristics and risk of bias among contributing studies. | 7-9 |
|  | 20b | Present results of all statistical syntheses conducted. If meta-analysis was done, present for each the summary estimate and its precision (e.g. confidence/credible interval) and measures of statistical heterogeneity. If comparing groups, describe the direction of the effect. | 7-9 |
|  | 20c | Present results of all investigations of possible causes of heterogeneity among study results. | 7-9 |
|  | 20d | Present results of all sensitivity analyses conducted to assess the robustness of the synthesized results. | 7-9 |
| Reporting biases | 21 | Present assessments of risk of bias due to missing results (arising from reporting biases) for each synthesis assessed. | 7-9 |
| Certainty of evidence | 22 | Present assessments of certainty (or confidence) in the body of evidence for each outcome assessed. | 7-9 |
| **DISCUSSION** | | |  |
| Discussion | 23a | Provide a general interpretation of the results in the context of other evidence. | 9 |
|  | 23b | Discuss any limitations of the evidence included in the review. | 11 |
|  | 23c | Discuss any limitations of the review processes used. | 11 |
|  | 23d | Discuss implications of the results for practice, policy, and future research. | 12 |
| **OTHER INFORMATION** | | |  |
| Registration and protocol | 24a | Provide registration information for the review, including register name and registration number, or state that the review was not registered. | 4 |
|  | 24b | Indicate where the review protocol can be accessed, or state that a protocol was not prepared. | 4 |
|  | 24c | Describe and explain any amendments to information provided at registration or in the protocol. | 4 |
| Support | 25 | Describe sources of financial or non-financial support for the review, and the role of the funders or sponsors in the review. | 12 |
| Competing interests | 26 | Declare any competing interests of review authors. | 12 |
| Availability of data, code and other materials | 27 | Report which of the following are publicly available and where they can be found: template data collection forms; data extracted from included studies; data used for all analyses; analytic code; any other materials used in the review. | 5-6 |

**Supplementary Table 3:** Keywords and search terms.

| **PICO** | **Subject Heading** | **Keywords** | |
| --- | --- | --- | --- |
| **RRT** | "Hospital Rapid Response Team"[Mesh] | rapid response team  medical emergency team  critical care outreach  patient at risk team  ICU outreach  deteriorating patient  clinical deterioration  patient deterioration  early warning score* | |
|  | ("rapid response team" OR "medical emergency team" OR "critical care outreach" OR "icu outreach" OR "patient at risk" OR "clinical deteriora*" OR "patient deteriora*" OR "deteriorating patient" OR "at risk patient" OR "early warning scor*" ) | | |
| **Sepsis** | "Sepsis"[Mesh] | Sepsis  Systemic inflammatory response syndrome  SIRS  Sequential organ failure assessment  Sepsis-related organ failure assessment  SOFA  qSOFA  Urosepsis | |
|  | ("sepsis" OR "septic" OR "systemic inflammatory response syndrome" OR "sirs" OR "sequential organ failure assessment" OR "sepsis related organ failure assessment" OR "sofa" OR "qsofa" OR "urosepsis") | | |
| **Infection** | "Infection"[Mesh] | | Infection  Pneumonia  Cellulitis  Abscess  Peritonitis |
|  | ("Infection" OR "cellulitis" OR "abscess" OR "pneumonia" OR "peritonitis" OR "bacteremia" OR "septicaemia") | | |

| PubMed | (("Hospital Rapid Response Team"[MeSH Terms] OR ("rapid response"[Title/Abstract] OR "medical emergency team"[Title/Abstract] OR "critical care outreach"[Title/Abstract] OR "icu outreach"[Title/Abstract] OR "patient at risk"[Title/Abstract] OR "clinical deteriora*"[Title/Abstract] OR "patient deteriora*"[Title/Abstract] OR "deteriorating patient"[Title/Abstract] OR "at risk patient"[Title/Abstract] OR "early warning scor*"[Title/Abstract])) AND ("Sepsis"[MeSH Terms] OR "Infections"[MeSH Terms] OR ((("Sepsis"[Title/Abstract] OR "septic"[Title/Abstract]) AND "infection"[Title/Abstract]) OR "systemic inflammatory response syndrome"[Title/Abstract] OR "SIRS"[Title/Abstract] OR "sequential organ failure assessment"[Title/Abstract] OR "sepsis related organ failure assessment"[Title/Abstract] OR "SOFA"[Title/Abstract] OR "qSOFA"[Title/Abstract] OR "urosepsis"[Title/Abstract] OR "cellulitis"[Title/Abstract] OR "abscess"[Title/Abstract] OR "pneumonia"[Title/Abstract] OR "Peritonitis"[Title/Abstract] OR "bacteremia"[Title/Abstract] OR "septicaemia"[Title/Abstract]))) AND ((humans[Filter]) AND (2015/1/1:3000/12/31[pdat])) AND (english[Filter]) | 1,932 |
| --- | --- | --- |
| Scopus | ( ( TITLE ( "rapid response team" OR "medical emergency team" OR "critical care outreach" OR "icu outreach" OR "patient at risk" OR "clinical deteriora*" OR "patient deteriora*" OR "deteriorating patient" OR "at risk patient" OR "early warning scor*" ) OR ABS ( "rapid response team" OR "medical emergency team" OR "critical care outreach" OR "icu outreach" OR "patient at risk" OR "clinical deteriora*" OR "patient deteriora*" OR "deteriorating patient" OR "at risk patient" OR "early warning scor*" ) ) ) AND ( ( TITLE ( "sepsis" OR "septic" OR "infection" OR "systemic inflammatory response syndrome" OR "sirs" OR "sequential organ failure assessment" OR "sepsis related organ failure assessment" OR "sofa" OR "qsofa" OR "urosepsis" OR "cellulitis" OR "abscess" OR "pneumonia" OR "peritonitis" OR "bacteremia" OR "septicaemia" ) OR ABS ( "sepsis" OR "septic" OR "infection" OR "systemic inflammatory response syndrome" OR "sirs" OR "sequential organ failure assessment" OR "sepsis related organ failure assessment" OR "sofa" OR "qsofa" OR "urosepsis" OR "cellulitis" OR "abscess" OR "pneumonia" OR "peritonitis" OR "bacteremia" OR "septicaemia" ) ) ) AND PUBYEAR > 2014 AND PUBYEAR < 2025 AND ( LIMIT-TO ( EXACTKEYWORD , "Human" ) OR LIMIT-TO ( EXACTKEYWORD , "Humans" ) OR EXCLUDE ( EXACTKEYWORD , "Nonhuman" ) ) AND ( LIMIT-TO ( LANGUAGE , "English" ) ) | 3,108 |
| Web of Science | **((TI=("Rapid Response Team" OR "medical emergency team" OR "critical care outreach" OR "icu outreach" OR "patient at risk" OR "clinical deteriora*" OR "patient deteriora*" OR "deteriorating patient" OR "at risk patient" OR "early warning scor*" ) OR AB=("Hospital Rapid Response Team" OR "rapid response" OR "medical emergency team" OR "critical care outreach" OR "icu outreach" OR "patient at risk" OR "clinical deteriora*" OR "patient deteriora*" OR "deteriorating patient" OR "at risk patient" OR "early warning scor*" ) AND (TI=("Sepsis" OR "septic" OR "infection" OR "systemic inflammatory response syndrome" OR "SIRS" OR "sequential organ failure assessment" OR "sepsis related organ failure assessment" OR "SOFA" OR "qSOFA" OR "urosepsis" OR "cellulitis" OR "abscess" OR "pneumonia" OR "Peritonitis" OR "bacteremia" OR "septicaemia")) OR AB=("Sepsis" OR "septic" OR "infection" OR "systemic inflammatory response syndrome" OR "SIRS" OR "sequential organ failure assessment" OR "sepsis related organ failure assessment" OR "SOFA" OR "qSOFA" OR "urosepsis" OR "cellulitis" OR "abscess" OR "pneumonia" OR "Peritonitis" OR "bacteremia" OR "septicaemia"))) AND (PY=("2015" OR "2016" OR "2017" OR "2018" OR "2019" OR "2020" OR "2021" OR "2022" OR "2023" OR "2024")) NOT ALL=(animal)** | 1,383 |
| The Cochrane Library | (("Hospital Rapid Response Team"[MeSH Terms] OR ("Early Warning Score"[MeSH Terms] OR (("Rapid Response Team" OR "medical emergency team" OR "critical care outreach" OR "icu outreach" OR "patient at risk" OR "clinical deterioration" OR "patient deterioration" OR "deteriorating patient" OR "at risk patient" OR "early warning score" OR "early warning scoring"):ti OR ("Rapid Response Team" OR "medical emergency team" OR "critical care outreach" OR "icu outreach" OR "patient at risk" OR "clinical deterioration" OR "patient deterioration" OR "deteriorating patient" OR "at risk patient" OR "early warning score" OR "early warning scoring"):ab) AND (("Sepsis"[MeSH Terms] OR "Sepsis" OR "septic" OR "infection" OR "systemic inflammatory response syndrome" OR "SIRS" OR "sequential organ failure assessment" OR "sepsis related organ failure assessment" OR "SOFA" OR "qSOFA" OR "urosepsis" OR "cellulitis" OR "abscess" OR "pneumonia" OR "Peritonitis" OR "bacteremia" OR "septicaemia"):ti OR ("Sepsis" OR "septic" OR "infection" OR "systemic inflammatory response syndrome" OR "SIRS" OR "sequential organ failure assessment" OR "sepsis related organ failure assessment" OR "SOFA" OR "qSOFA" OR "urosepsis" OR "cellulitis" OR "abscess" OR "pneumonia" OR "Peritonitis" OR "bacteremia" OR "septicaemia"):ab)  with Cochrane Library publication date from Jan 2015 to Dec 2024  Limiters: Trials; English | 407 |
| CINAHL | ((MH "Rapid Response Team") OR (MH "Early Warning Score") OR TI ("Rapid Response Team" OR "medical emergency team" OR "critical care outreach" OR "icu outreach" OR "patient at risk" OR "clinical deteriora*" OR "patient deteriora*" OR "deteriorating patient" OR "at risk patient" OR "early warning scor*" ) OR AB ("Rapid Response Team" OR "medical emergency team" OR "critical care outreach" OR "icu outreach" OR "patient at risk" OR "clinical deteriora*" OR "patient deteriora*" OR "deteriorating patient" OR "at risk patient" OR "early warning scor*" )) AND ((MH "Sepsis") OR (MH "Infection") OR TI ( "Sepsis" OR "septic" OR "infection" OR "systemic inflammatory response syndrome" OR "SIRS" OR "sequential organ failure assessment" OR "sepsis related organ failure assessment" OR "SOFA" OR "qSOFA" OR "urosepsis" OR "cellulitis" OR "abscess" OR "pneumonia" OR "Peritonitis" OR "bacteremia" OR "septicaemia" ) OR AB ( "Sepsis" OR "septic" OR "infection" OR "systemic inflammatory response syndrome" OR "SIRS" OR "sequential organ failure assessment" OR "sepsis related organ failure assessment" OR "SOFA" OR "qSOFA" OR "urosepsis" OR "cellulitis" OR "abscess" OR "pneumonia" OR "Peritonitis" OR "bacteremia" OR "septicaemia" )  **Limiters** - Publication Date: 20150101-20241231; Human  **Narrow by Language:**- english | 378 |
| Embase | ('rapid response team'/exp OR 'rapid response team':ab,ti OR 'medical emergency team':ab,ti OR 'critical care outreach':ab,ti OR 'icu outreach':ab,ti OR 'patient at risk':ab,ti OR 'clinical deteriora*':ab,ti OR 'patient deteriora*':ab,ti OR 'deteriorating patient':ab,ti OR 'at risk patient':ab,ti OR 'early warning scor*':ab,ti) AND ('sepsis'/exp OR 'infection'/exp OR 'sepsis':ab,ti OR 'septic':ab,ti OR 'infection':ab,ti OR 'systemic inflammatory response syndrome':ab,ti OR 'sirs':ab,ti OR 'sequential organ failure assessment':ab,ti OR 'sepsis related organ failure assessment':ab,ti OR 'sofa':ab,ti OR 'qsofa':ab,ti OR 'urosepsis':ab,ti OR 'cellulitis':ab,ti OR 'abscess':ab,ti OR 'pneumonia':ab,ti OR 'peritonitis':ab,ti OR 'bacteremia':ab,ti OR 'septicaemia':ab,ti) AND [humans]/lim AND [2015-2024]/py AND [english]/lim AND 'article'/it | 2,027 |

**Supplementary Table 4:** Sepsis-related RRT reviews.

| **First Author, Year of Publication** | **Total RRTs** | **Number of sepsis - related RRTs** |
| --- | --- | --- |
| **RRTs Non-exclusive for Sepsis** | | |
| Kim 2021 | 5277 | 694 |
| Gershkovich 2019 | 401 | 318 |
| Choi 2021 | 18961 | 976 |
| White 2016 | 1151 | 137 |
| Robertson 2021 | 266 | 141 |
| Kim 2017 | 1219 | 414 |
| Nolan 2024 | 2648 | 84 |
| Lee 2015 | 525 | 213 |
| Mullins 2016 | 795 | 153 |
| Ramos 2020 | 213 | 23 |
| Orosz 2020 | 55084 | 17799 |
| Zhang 2021 | 605 | 8 |
| Gupta 2021 | 31392 | 18490 |
| Azraai 2021 | 350 | 70 |
| Baek 2017 | 69 | 8 |
| Messmer 2022 | 4068 | 743 |
| LeGuen 2018 | 258 | 127 |
| Lee 2022 | 1096 | 960 |
| Jung 2016 | 564 | 142 |
| Saku 2020 | 65 | 4 |
| Smith 2017 | 937 | 99 |
| **RRTs exclusive for Sepsis** | | |
| Al-Qahtani 2019 | 315 | 157 |
| Ludikhuize 2023 | 1940 | 485 |
| Fernando 2018 | 6023 | 1708 |
| Boulos 2017 | 4496 | 970 |
| Cross 2015 | 358 | 159 |
| **TOTAL** | **139,076** | **45082 (32.4%)** |

**Supplementary Table 5:** Primary and Secondary Outcomes.

| **Outcome** | **Studies with all causes of RRT calls** | | | | **Studies with exclusively sepsis-related RRT calls** | | |
| --- | --- | --- | --- | --- | --- | --- | --- |
|  | **Studies** | **Point Estimate (95%CI)** | **GRADE Certainty** | **Studies** | | **Point Estimate (95%CI)** | **GRADE Certainty** |
| **Primary outcome** | | | | | | | |
| Sepsis RRTs | 21 | 21.8% (95%CI: 12.8%-34.5%) | Moderate^1^ | 5 | | 32.7% (95%CI: 23.9%-43.0%) | Moderate^1^ |
| **Secondary outcomes** | | | | | | | |
| In-hospital Mortality | 4 | 14.2% (95%-CI: 6.9%-26.8%) | High^2^ | 7 | | 11.2% (95%CI: 4.4%-24.9%) | High^2^ |
| Hospital length of stay | 13 | 18.4 (95%CI: 12.8-24.1) | High | 5 | | 16.6 (95%CI: 14.1-19.2) | Moderate^1^ |
| ^1^ Downgrade for inconsistency  ^2^ Downgrade for imprecision  Abbreviations: RRT – Rapid Response team, CI – confidence interval, GRADE - Grading of Recommendations, Assessment, Development, and Evaluations | | | | | | | |

**Supplementary Table 6:** Risk of bias assessment using Newcastle-Ottawa Scale.

| **Study ID** | **Represent-ativeness of the Exposed Cohor** | **Selection of the Non-Exposed Cohort** | **Ascertainment of Exposure** | **Demonstration that Outcome of Interest was not present at start of study** | **Comparability of Cohorts on the basis of the Design or Analysis** | **Assessment of Outcome** | **Was Follow-Up Long Enough for Outcomes to Occur** | **Adequacy of Follow Up of Cohorts** | **Score** | **NOS quality** |
| --- | --- | --- | --- | --- | --- | --- | --- | --- | --- | --- |
| Kim 2021 | Yes | Yes | Yes | Yes | Yes | Yes | Yes | Yes | 9 | Good |
| Gershkovich 2019 | Yes | Yes | Yes | Unsure | Yes | Yes | Yes | Yes | 8 | Good |
| Al-Qahtani 2019 | Yes | Yes | Yes | Yes | No | Yes | Yes | Yes | 7 | Good |
| Choi 2021 | Yes | Yes | Yes | Unsure | Yes | Yes | Yes | Yes | 8 | Good |
| White 2016 | Unsure | Yes | Yes | Unsure | Unsure | Yes | Yes | Yes | 5 | Fair |
| Robertson 2021 | Yes | Yes | Yes | Yes | Yes | Yes | Yes | Yes | 9 | Good |
| Kim 2017 | Yes | Yes | Yes | Unsure | Yes | Yes | Yes | Yes | 8 | Good |
| Ludikhuize 2023 | Unsure | No | No | Yes | No | Yes | Yes | Yes | 4 | Fair |
| Nolan 2024 | Yes | Yes | Yes | Unsure | Yes | Yes | Yes | Yes | 8 | Good |
| Lee 2015 | No | Yes | Yes | Unsure | Yes | Unsure | Unsure | Yes | 5 | Fair |
| Mullins 2016 | Yes | Yes | Yes | Unsure | Unsure | No | Yes | Yes | 5 | Fair |
| Ramos 2020 | Yes | Yes | Yes | Yes | Yes | Yes | Yes | Yes | 9 | Good |
| Orosz 2020 | Yes | Yes | Yes | Yes | Yes | Yes | Yes | Yes | 9 | Good |
| Zhang 2021 | Yes | Yes | Yes | Yes | Yes | Yes | Yes | Yes | 9 | Good |
| Gupta 2021 | Yes | Yes | Yes | Yes | Unsure | Yes | Yes | Yes | 7 | Good |
| Azraai 2021 | No | Unsure | Yes | Yes | Unsure | Yes | Yes | Yes | 5 | Fair |
| Baek 2017 | No | Unsure | Yes | Yes | Yes | Yes | Yes | Yes | 7 | Good |
| Messmer 2022 | Yes | Yes | Yes | Yes | Yes | Yes | Yes | Yes | 9 | Good |
| Fernando 2018 | Yes | Yes | Yes | No | Unsure | Yes | Yes | Yes | 6 | Fair |
| Boulos 2017 | Yes | Yes | Yes | Unsure | Yes | Yes | Yes | Yes | 8 | Good |
| LeGuen 2018 | Yes | Yes | Yes | Yes | Yes | Yes | Unsure | Yes | 8 | Good |
| Cross 2015 | Yes | Yes | Yes | Yes | Unsure | Yes | Yes | Yes | 7 | Good |
| Lee 2022 | Yes | Yes | Yes | Unsure | Unsure | Unsure | Yes | Yes | 5 | Fair |
| Jung 2016 | Yes | Yes | Yes | Yes | Yes | Yes | Yes | Yes | 9 | Good |
| Saku 2020 | No | Yes | Yes | Yes | No | Yes | Unsure | Yes | 5 | Fair |
| Smith 2017 | Yes | Yes | Yes | Yes | Yes | Yes | Yes | Yes | 9 | Good |

**Supplementary Table 7:** Rapid Response Team Triggers.

| **First Author,  Year of Publication** | **Total RRTs** | **Airway concern** | **Respiratory rate (high or low)** | **Hypoxia despite O_2_ therapy** | **Bradycardia or Tachycardia** | **Hypotension** | **Altered GCS** | **Clinical concern** | **2 or more triggers** |
| --- | --- | --- | --- | --- | --- | --- | --- | --- | --- |
| **Studies including all causes of RRT calls** | | | | | | | | | |
| Kim 2021 | 5277 | - | - | - | - | - | - | - | - |
| Gershkovich 2019 | 401 | 8 | 124 | 49 | 68 | 56 | 48 | 52 | - |
| Choi 2021 | 18961 | - | - | - | - | - | - | - | - |
| White 2016 | 1151 | 0 | 140 | 227 | 176 | 339 | 315 | 175 | 217 |
| Robertson 2021 | 266 | 0 | 91 | 28 | 86 | 47 | 31 | 43 | - |
| Kim 2017 | 1219 | - | - | - | - | - | - | - | - |
| Nolan 2024 | 2648 | 48 | 364 | 249 | 324 | 393 | 496 | 373 | - |
| Lee 2015 | 525 | 52 | 237 | 245 | 268 | 222 | 78 | 19 | - |
| Mullins 2016 | 795 | 0 | 171 | 0 | 209 | 82 | 200 | 40 | - |
| Ramos 2020 | 213 | 0 | 0 | 0 | 0 | 213 | 0 | 0 | - |
| Orosz 2020 | 55084 | - | - | - | - | - | - | - | - |
| Zhang 2021 | 605 | 7 | 68 | 43 | 157 | 226 | 52 | 21 | - |
| Gupta 2021 | 31392 | 314 | 1570 | 4081 | 3139 | 4709 | 3767 | 11615 | - |
| Azraai 2021 | 350 | 0 | - | 57 | 140 | 114 | 225 | 56 | - |
| Baek 2017 | 69 | - | - | - | - | - | - | - | - |
| Messmer 2022 | 4068 | 0 | 44 | 776 | 0 | 0 | 0 | 1192 | - |
| LeGuen 2018 | 258 | - | - | - | - | - | - | - | - |
| Lee 2022 | 1096 | - | - | - | - | - | - | - | - |
| Jung 2016 | 564 | 0 | 99 | 97 | 35 | 79 | 76 | 36 | - |
| Saku 2020 | 65 | 2 | 5 | 24 | 7 | 15 | 22 | 12 | 24 |
| Smith 2017 | 937 | 15 | 150 | 163 | 145 | 269 | 228 | 116 | - |
| **TOTAL** | **125944** | **446** | **3063** | **6039** | **4754** | **6764** | **5538** | **13750** | **241** |
| **Percentage** |  | **1.0%** | **7.0%** | **13.7%** | **10.8%** | **15.4%** | **12.6%** | **31.3%** | **19.8%** |
| **Studies including exclusively sepsis-related RRT calls** | | | | | | | | | |
| Al-Qahtani 2019 | 315 | 1 | 93 | 47 | 66 | 77 | 54 | 13 | - |
| Ludikhuize 2023 | 1940 | 43 | 377 | 209 | 548 | 391 | 141 | 114 | - |
| Fernando 2018 | 6023 | 37 | 586 | 0 | 244 | 315 | 202 | 191 | - |
| Boulos 2017 | 4496 | 0 | 148 | 109 | 137 | 144 | 49 | 59 | - |
| Cross 2015 | 358 | 0 | 213 | 0 | 273 | 0 | 0 | 0 | - |
| **TOTAL** | **11677** | **81** | **1417** | **365** | **1268** | **927** | **446** | **377** | **0** |
| **Percentage** |  | **0.7%** | **12.1%** | **3.1%** | **10.9%** | **7.9%** | **3.8%** | **3.2%** | - |
|  | | | | | | | | | |
| **Overall (%)** | **139,076** | **(1.0%)** | **(8.5%)** | **(12.2%)** | **(11.4%)** | **(14.6%)** | **(11.4%)** | **(26.8%)** | **(19.8%)** |
|  | | | | | | | | | |
| **p-value*** | **-** | **<0.001** | **<0.001** | **<0.001** | 0.06 | **<0.001** | **<0.001** | **<0.001** | **<0.001** |
| * Comparison between RRTs non-exclusive vs. exclusive for sepsis | | | | | | | | | |

**Supplementary Table 8:** Presumed or confirmed source of sepsis.

| **First Author, Year of Publication** | **Total RRTs** | **Number of sepsis RRTs** | **Source of infection** | | | | | |
| --- | --- | --- | --- | --- | --- | --- | --- | --- |
|  |  |  | **Chest** | **Skin** | **Urinary** | **CNS** | **Intra-abdominal** | **Others** |
| **Studies including all causes of RRT calls** | | | | | | | | |
| Kim 2021 | - | - | - | - | - | - | - | - |
| Gershkovich 2019 | 401 | 318 | 151 | 10 | 35 | 6 | 89 | 27 |
| Choi 2021 | 18961 | 976 | 261 | 0 | 58 | 0 | 434 | 0 |
| White 2016 | 266 | 141 | 34 | - | - | - | - | - |
| Robertson 2021 | - | - | - | - | - | - | - | - |
| Kim 2017 | - | - | - | - | - | - | - | - |
| Nolan 2024 | - | - | - | - | - | - | - | - |
| Lee 2015 | - | - | - | - | - | - | - | - |
| Mullins 2016 | - | - | - | - | - | - | - | - |
| Ramos 2020 | - | - | - | - | - | - | - | - |
| Orosz 2020 | - | - | - | - | - | - | - | - |
| Zhang 2021 | - | - | - | - | - | - | - | - |
| Gupta 2021 | - | - | - | - | - | - | - | - |
| Azraai 2021 | - | - | - | - | - | - | - | - |
| Baek 2017 | - | - | - | - | - | - | - | - |
| Messmer 2022 | - | - | - | - | - | - | - | - |
| LeGuen 2018 | 258 | 127 | 77 | 0 | 10 | 0 | 29 | 11 |
| Lee 2022 | - | - | - | - | - | - | - | - |
| Jung 2016 | - | - | - | - | - | - | - | - |
| Saku 2020 | - | - | - | - | - | - | - | - |
| Smith 2017 | 937 | 99 | 19 | 0 | 17 | 0 | 16 | 47 |
| **TOTAL** | **20823** | **1661** | **542** | **10** | **120** | **6** | **584** | **85** |
|  |  | **8.0%** | **32.6%** | **0.7%** | **7.9%** | **0.4%** | **38.4%** | **5.6%** |
| **Studies including exclusively sepsis-related RRT calls** | | | | | | | | |
| Al-Qahtani 2019 | 315 | 157 | 33 | 0 | 18 | 0 | 21 | 25 |
| Ludikhuize 2023 | 1940 | 485 | - | - | - | - | - | - |
| Fernando 2018 | 6023 | 1708 | 811 | 68 | 235 | 42 | 450 | 102 |
| Boulos 2017 | 4496 | 970 | 218 | 0 | 123 | 0 | 0 | 0 |
| Cross 2015 | 358 | 159 | 86 | 10 | 26 | 0 | 38 | 48 |
|  | | | | | | | | |
| **TOTAL** | **11677** | **3479** | **1148** | **78** | **402** | **42** | **471** | **175** |
| **Percentage** |  | **29.8%** | **38.3%** | **2.6%** | **13.4%** | **1.4%** | **15.7%** | **5.8%** |
|  | | | | | | | | |
| **p-value*** |  | **<0.001** | **<0.001** | **<0.001** | **<0.001** | **<0.001** | **<0.001** | 0.08 |
| * Comparison between RRTs non-exclusive vs. exclusive for sepsis | | | | | | | | |

**Supplementary Table 9:** Hospital vs Community acquired infections as observed during rapid response team reviews.

| **First Author, Year of Publication** | **Total RRTs** | **Hospital acquired** | **Community acquired** |
| --- | --- | --- | --- |
| **Studies including all causes of RRT calls** | | | |
| Nolan 2024 | 2648 | - | 84 |
| **Studies including exclusively sepsis-related RRT calls** | | | |
| Boulos 2017 | 4496 | 123 | 382 |
| Cross 2015 | 358 | 91 | 67 |
| **TOTAL** |  | **214** | **533** |
| **Denominator** |  | 1129 | 1203 |
| **Percentage** |  | **19.0%** | **44.3%** |

**Supplementary Table 10:** Interventions during rapid response team reviews.

| **First Author, Year of Publication** | **Total Number of RRTs** | **New or change in antibiotics** | **iv fluids** | **Transfusion** | **Vasopressors** | **Ventilatory support** | **Expeditated Source Control** | **Steroids** | **Diuretics** | **Antiarrhythmics** | **Dtic Imaging** | **Meds changed/ withheld** | **ECMO** | **ACLS** | **Observation / No Treatment** |
| --- | --- | --- | --- | --- | --- | --- | --- | --- | --- | --- | --- | --- | --- | --- | --- |
| **Studies including all causes of RRT calls** | | | | | | | | | | | | | | | |
| Kim 2021 | 5277 | - | - | - | - | - | - | - | - | - | - | - | - | - | - |
| Gershkovich 2019 | 401 | - | - | - | - | - | - | - | - | - | - | - | - | - | - |
| Choi 2021 | 18961 | 940 | 885 | 231 | 976 | 343 | 169 | 266 | - | - | - | - | - | - | - |
| White 2016 | 1151 | - | 341 | - | 39 | - | - | - | - | - | - | - | - | - | - |
| Robertson 2021 | 266 | 75 | 101 | - | - | 20 | - | - | - | - | 164 | - | - | - | - |
| Kim 2017 | 1219 | - | - | - | - | - | - | - | - | - | - | - | - | - | - |
| Nolan 2024 | 2648 | 23 | - | - | - | - | - | - | - | - | - | - | - | - | - |
| Lee 2015 | 525 | - | - | - | 246 | 216 | - | - | - | - | - | - | - | - | - |
| Mullins 2016 | 795 | - | 324 | - | - | 8 | - | - | - | - | - | - | - | - | - |
| Ramos 2020 | 213 | 10 | 173 | 30 | 9 | - | - | - | - | - | - | 40 | - | - | 21 |
| Orosz 2020 | 55084 | - | - | - | - | 13982 | - | - | - | - | - | - | - | - | - |
| Zhang 2021 | 605 | - | - | - | - | 97 | - | - | - | - | - | - | - | - | - |
| Gupta 2021 | 31392 | - | - | - | - |  | - | - | - | - | - | - | - | - | - |
| Azraai 2021 | 350 | 62 | 94 | - | 7 | 21 | - | - | - | - | - | 75 | - | - | 60 |
| Baek 2017 | 69 | - | 48 | 48 | 15 | 16 | - | - | - | - | - |  | 3 | - | - |
| Messmer 2022 | 4068 | - | - | - | - | - | - | - | - | - | - | - | - | - | - |
| LeGuen 2018 | 258 | - | - | - | - | - | - | - | - | - | - | - | - | - | - |
| Lee 2022 | 1096 | - | - | - | 83 | 315 | - | - | - | - | - | - | 3 | 41 |  |
| Jung 2016 | 564 | 43 | 159 | - | 21 | 48 | - | - | - | - | - | - | - | - | - |
| Saku 2020 | 65 | - | 19 | - | 10 | 6 | - | - | - | - | - | - | - | - | - |
| Smith 2017 | 937 | 63 | - | 65 | 48 | 16 | - | 65 | 84 | 82 | - | 50 | - | - | - |
| **Studies including exclusively sepsis-related RRT calls** | | | | | | | | | | | | | | | |
| Al-Qahtani 2019 | 315 | 371 | - | - | - | - | - | - | - | - | - | - | - | - | - |
| Ludikhuize 2023 | 1940 | 190 | - | - | - | - | - | - | - | - | - | - | - | - | - |
| Fernando 2018 | 6023 | - | - | - | 525 | 378 | - | - | - | - | - | - | - | - | - |
| Boulos 2017 | 4496 | 593 | - | - | 118 | 112 | - | - | - | - | - | - | - | - | - |
| Cross 2015 | 358 | 76 | - | - | - | - | - | - | - | - | - | - | - | - | - |

**Supplementary Table 11:** Disposition at the end of a rapid response team review.

| **First Author, Year of Publication** | **Total number of RRTs** | **Number sepsis-related RRTs** | **Stay on ward** | **Transferred to ICU or HDU** | **Transferred to CCU** | **Emergency Surgery** | **Other disposition** | **Died at MET** |
| --- | --- | --- | --- | --- | --- | --- | --- | --- |
| **Studies including all causes of RRT calls** | | | | | | | | |
| Kim 2021 | 5277 | - | - | - | - | - | - | - |
| Gershkovich 2019 | 401 | 318 | - | 145 | - | - | - | - |
| Choi 2021 | 18961 | 976 | - | 578 | - | 169 | - | - |
| White 2016 | 1151 | 141 | 930 | 128 | 39 | 38 | - | - |
| Robertson 2021 | 266 | - | 198 | 64 | - | - | - | - |
| Kim 2017 | 1219 | - | 751 | 468 | - | - | - | - |
| Nolan 2024 | 2648 | - | - |  | - | - | - | - |
| Lee 2015 | 525 | - | - | 525 | - | - | - | - |
| Mullins 2016 | 795 | - | - |  | - | - | - | - |
| Ramos 2020 | 213 | - | - | 10 | - | - | - | - |
| Orosz 2020 | 55084 | - | - | 17799 | - | - | - | - |
| Zhang 2021 | 605 | - | - | 97 | - | - | - | - |
| Gupta 2021 | 31392 | - | 27311 | 2197 | - | - | - | - |
| Azraai 2021 | 350 | - | 248 | 59 | 17 | 9 | 17 | - |
| Baek 2017 | 69 | - | 48 | 21 | - | - | - | - |
| Messmer 2022 | 4068 | - | 1993 | 1871 | - | - | 32 | - |
| LeGuen 2018 | 258 | 127 | 100 | 27 | - | - | - | - |
| Lee 2022 | 1096 | - | 707 | 389 | - | - | - | - |
| Jung 2016 | 564 | - | - | 187 | - | - | - | 19 |
| Saku 2020 | 65 | - | 35 | 29 | - | - | - | - |
| Smith 2017 | 937 | 99 | 721 | 110 | - | 26 | 16 | 6 |
| **Studies including exclusively sepsis-related RRT calls** | | | | | | | | |
| Al-Qahtani 2019 | 315 | 157 | 171 | 114 | - | - | - | - |
| Ludikhuize 2023 | 1940 | 485 | 439 | 40 | - | 1 | 5 | - |
| Fernando 2018 | 6023 | 1708 | - | 600 | - | - | - | - |
| Boulos 2017 | 4496 | 970 | - | 200 | - | - | - | 99 |
| Cross 2015 | 358 | 159 | - | - | - | - | - | - |
